# False Lumen Stenting to the Acute Occlusive Carotid Artery Dissection Combined With Intracranial Acute Embolic Stroke: A Case Report and Literature Review

**DOI:** 10.7759/cureus.75317

**Published:** 2024-12-08

**Authors:** Takahiro Morita, Sakura Akitaya, Ryu-ichi Munakata, Atsushi Saito

**Affiliations:** 1 Department of Neurosurgery, Hirosaki University Graduate School of Medicine, Hirosaki, JPN

**Keywords:** acute ischemic stroke (ais), carotid stenting, false lumen, spontaneous internal carotid artery dissection, tandem lesion

## Abstract

Tandem occlusion due to acute cervical carotid artery dissection should be promptly treated with thrombectomy for reperfusion. If the cervical lesion has reached severe stenosis or complete occlusion, balloon angioplasty and, in certain cases, carotid artery stenting should be performed before thrombectomy for the intracranial lesion. Angioplasty or stent placement is performed in the true lumen, but securing the placement is challenging when the true lumen cannot be determined. In contrast, stenting in the false lumen of a carotid artery dissection is considered contraindicated. Although reports on a few similar cases have been published, no obvious complications are known, and the actual risks and outcomes remain unclear.

We report the case of a 49-year-old woman with acute ischemic stroke who had tandem occlusion of the cervical internal carotid and middle cerebral arteries due to acute cervical dissection. The cervical lesion was completely occluded with no true lumen, and securing the true lumen proved extremely difficult. Therefore, we performed intracranial thrombectomy via the false lumen, followed by carotid artery stenting from the distal to the proximal true lumen via the false lumen. Six months later, follow-up examinations revealed no obvious complications.

Our literature review identified only three reports of stenting in the false lumen of an acute carotid artery dissection, and no apparent complications were reported in any of these cases. Furthermore, the technique of recanalization through the false lumen is well established in chronic total occluded lesions of coronary or peripheral arteries when the true lumen cannot be secured. Therefore, access to the intracranial artery via the false lumen may be acceptable in situations of simultaneous intracranial arterial occlusion requiring rapid recanalization where securing a true lumen is challenging.

## Introduction

Cervical carotid artery dissection (CAD) can be caused by endogenous factors such as hypertension, ultrastructural connective tissue abnormalities in the artery, genetic disease or infection, as well as by high-energy trauma such as a car accident or minor trauma such as chiropractic as in vertebral artery dissection. The typical symptoms include neck pain, headache, Horner’s syndrome, and ischemia [1,2]. Ischemia is said to be the cause of approximately 20% of cerebral infarctions in young patients and often requires urgent revascularization procedures [1,3-6]. Carotid artery stenting (CAS) is mainly performed when the lesion is located only in the neck, and satisfactory results have been reported [4]. However, tandem lesions, caused by thromboembolism in major intracranial arteries secondary to cervical CAD, often require simultaneous treatment of both lesions [6].

The standard treatment for CAD involves securing the true lumen and dilating the lesion. However, if the dissection is completely occluded and no true lumen has been delineated, or the lumen is visible but so severely damaged that the true and false lumens cannot be distinguished, securing the true lumen may prove challenging. This would delay the reopening of the intracranial obstruction and worsen the outcome [7]. It is possible to reach intracranial vessels via the false lumen; however, false lumen dilatation may lead to vessel rupture or acute re-occlusion [4,8]. Nonetheless, there have been few reports on carotid stenting of the false lumen in cervical CAD, and the actual risks and outcomes remain unclear.

In this study, we report a case of complete occlusion of the cervical artery and hyperacute embolic infarction of the main intracranial artery due to cervical internal carotid artery (ICA) dissection. The false lumen was stented, and the intracranial occlusion reopened without complications.

## Case presentation

**Figure 1:**
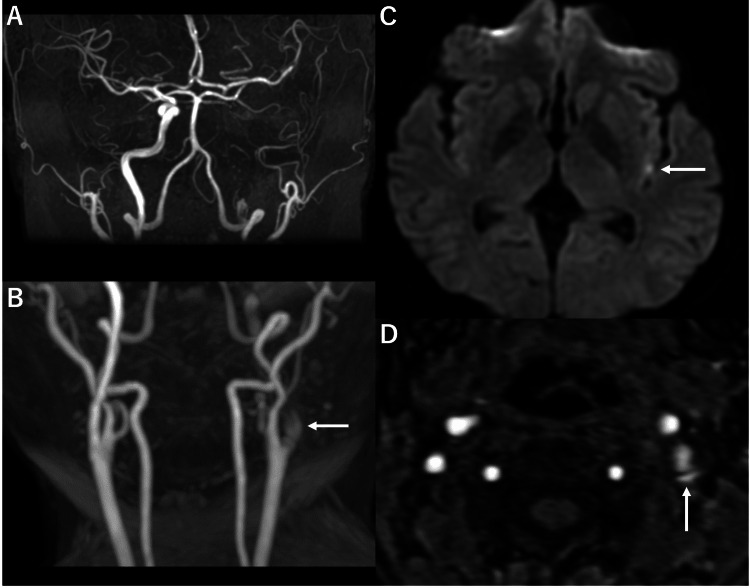
Initial magnetic resonance imaging performed at the neurosurgical clinic. A: Anterior-posterior (A-P) view of the head on magnetic resonance angiography (MRA). B: A-P view of the cervical MRA scan. The arrow indicates the occluded left internal carotid artery (ICA). C: Diffusion-weighted image. The arrow indicates asymptomatic acute infarctions. D: Source image of cervical MRA. The arrow indicates the intimal flap in the ICA, which is suspected to be a carotid artery dissection.

The patient was a 49-year-old woman who visited the neurosurgical clinic because of a persistent headache from the time of waking on the day of the visit. At the time of her visit, no abnormal neurological findings were observed. Head and neck magnetic resonance imaging (MRI) showed a slight diffusion-weighted high-signal lesion in the left insular cortex. In contrast, magnetic resonance angiography showed occlusion of the left ICA and intimal flap, suspected of a CAD. The left anterior cerebral artery and middle cerebral artery (MCA) were well delineated in other vessels via the anterior communicating artery and left posterior communicating artery (Figure 1).

The patient was referred to our hospital for further examination and treatment. Before the examination, the patient had right hemiplegia and total aphasia. On cerebral angiography, the left ICA was completely occluded from its origin, and the MCA, observed on MRI, was no longer visible.

**Figure 2:**
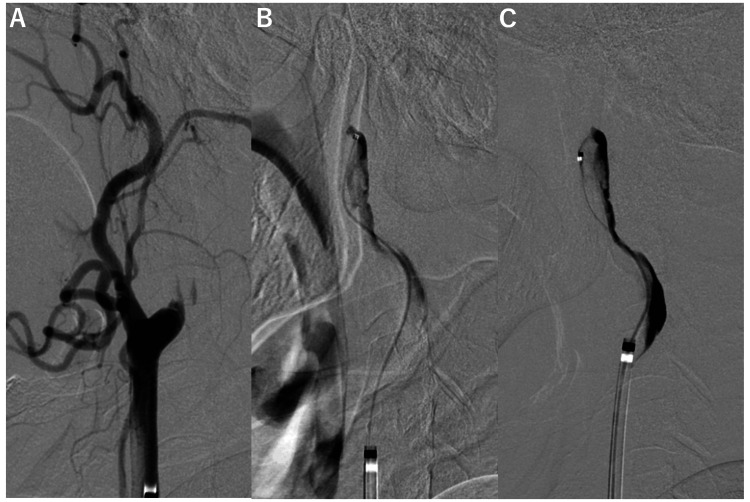
Microcatheter angiography during passage to the occluded cervical internal carotid artery (ICA). A: Angiogram showing the guiding catheter before advancing the micro guidewire/catheter. B, C: Serial angiogram of the microcatheter in the occluded ICA. The contrast media did not flow out into the intracranial ICA, but instead flowed back into the false lumen.

Therefore, we performed endovascular treatment as reperfusion therapy for hyperacute ischemic stroke. A balloon-guiding catheter was advanced to the origin of the left ICA and aspirated while blocking the origin; the occlusion site was visible slightly at the periphery, and an intimal flap was observed inside the occlusion site (Figure 2A). The patient was diagnosed with CAD. Because the location of the true lumen was not visible, the micro guidewire/catheter was advanced to the ICA, and the area with the least resistance was explored with the wire. The catheter was gradually advanced to the periphery; however, advancement was no longer possible proximal to the cranial entry point. Angiography via the microcatheter showed that the contrast media did not flow into the intracranial ICA; instead, it flowed back through what appeared to be a false lumen into the external carotid artery (ECA) (Figures 2B, 2C).

**Figure 3:**
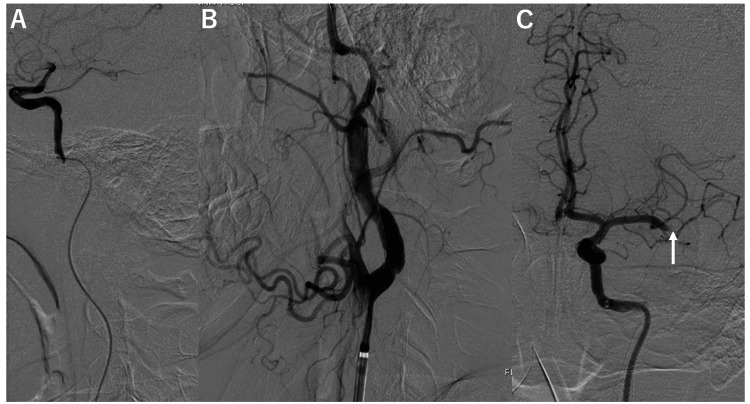
Intraoperative left carotid arteriogram after passing through the false lumen of a cervical internal carotid dissection. A: Lateral view of the microcatheter angiogram after passing through the false lumen and securing the true lumen in the intracranial portion. B: Lateral view of the guiding catheter placed at the cervical portion after balloon angioplasty from the distal portion of the true lumen via the false lumen in the lesion to the proximal true lumen. C: Anterior-posterior view of the intracranial angiogram after reopening the cervical internal carotid artery. The arrow indicates the occluded middle cerebral artery due to an artery-to-artery embolism.

The wire/catheter was thought to be in the false lumen; however, the entrance to the true lumen could not be confirmed, and advancing the catheter back into the true lumen was considered challenging. Therefore, we continued to explore a possible passage; eventually, we passed the catheter into the true lumen at the cranial entry point and secured the distal true lumen (Figure 3A). To reopen the occluded ICA, balloon angioplasty using a 3-mm-diameter balloon catheter was performed from the distal true lumen to the true lumen of the proximal common carotid artery via the false lumen (Figure 3B). Intracranial angiography revealed that the M2 inferior trunk of the left MCA was occluded at its origin (Figure 3C), suggesting that an artery-to-artery embolism occurred when the symptoms worsened.

**Figure 4:**
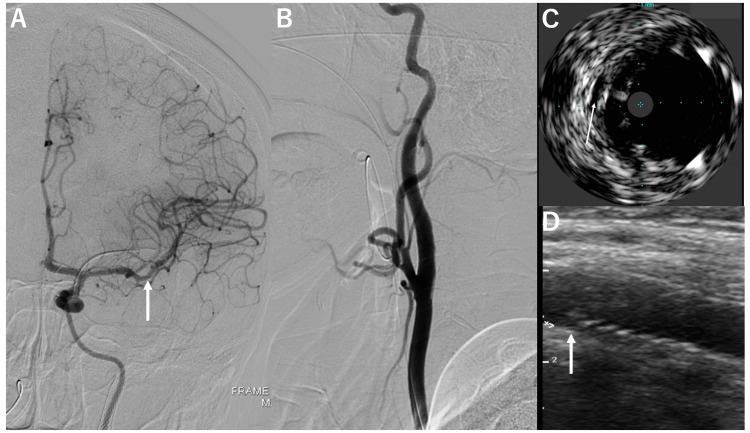
Final angiogram after treatment and postoperative imaging. A: Anterior-posterior view of the intracranial left internal carotid artery. The arrow indicates the reopened middle cerebral artery. B: Lateral view of the cervical carotid artery. C: Intravascular ultrasound image. D: Trans-surface carotid artery ultrasonogram. Arrows in C and D indicate the lumen outside the stent, and the true lumen is suspected.

Thrombectomy was performed using the combined technique, and thrombolysis in cerebral infarction grade 2b reperfusion was performed in a single procedure (Figure 4A). The onset-to-reperfusion time was 193 minutes. The cervical carotid artery was stented using two Wallstents (Boston Scientific, Marlborough, MA, USA) from the distal to the proximal true lumen (Figure 4B). Postoperative angiography revealed no abnormalities. Intravascular ultrasound (IVUS) scanning showed a lumen without blood flow outside the stent, which was thought to depict the true lumen (Figure 4C). Postoperatively, right hemiplegia improved, and rehabilitation was performed at another hospital for residual aphasia. Trans-surface carotid ultrasonography performed on postoperative day seven showed a lumen without blood flow outside the stent. However, no other abnormalities were noted (Figure 4D).

**Figure 5:**
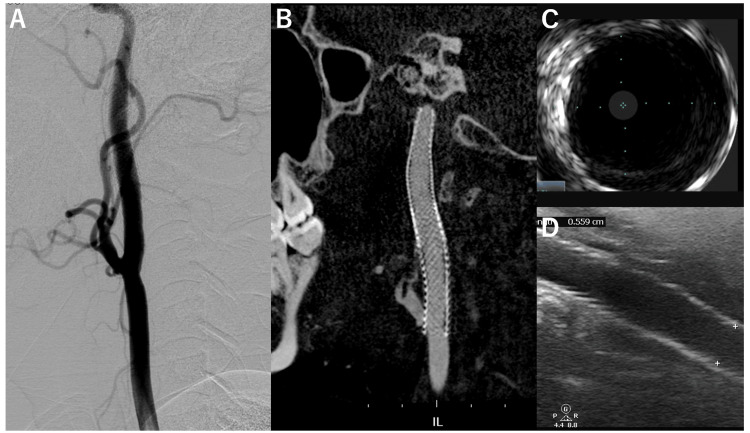
Imaging six months after the treatment. A: Lateral view of the left carotid artery. B: Lateral view in three-dimensional digital subtraction angiography of the left cervical carotid artery. C: Intravascular ultrasound of the left carotid artery. D: Trans-surface ultrasound image of the left carotid artery. No abnormalities were observed in these examinations.

Precisely 6 months after the treatment, the patient had moderate motor aphasia, with a modified Rankin scale (mRS) score of 1. No further abnormalities, such as restenosis of the lesion or reopening of the true lumen, were observed on imaging (Figure 5).

## Discussion

We reported a case of acute cerebral infarction in a tandem lesion with occlusive dissection of the cervical ICA and intracranial arterial embolism. Because the true lumen of the CAD was not delineated and passage was difficult, angioplasty of the false lumen, thrombectomy for intracranial artery occlusion, and CAS in the false lumen were performed in a single stage, with relatively good outcomes. To our knowledge, this is the fourth case of CAS in the false lumen and the first case showing that no complications from false lumen development occurred after the follow-up, including imaging up to the chronic phase.

In patients with intracranial occlusion associated with acute CAD, reopening the intracranial artery occlusion as quickly as possible contributes to a good outcome [9]. If stenosis associated with CAD is not severe and intracranial lesions can be accessed in one stage, the priority is to reopen intracranial lesions [7]; however, if cervical stenosis is severe or completely occluded and no access route has been delineated, the cervical lesion should be recanalized before treating the intracranial lesion [6].

When stenting a CAD, the standard technique is to secure the true lumen and deploy the stent; manipulating the false lumen is considered undesirable. Early reports of stenting for CAD indicate that the procedure was terminated because the true lumen could not be secured [10]. Thus, securing the true lumen is considered a prerequisite for endovascular treatment of CAD. Edgell et al. stated in their report that this was required “to prevent perforation,” and did not report any technical failures [4].

However, most reports [3,5,11-13] do not specifically describe the method used for securing the true lumen or only report “careful” passage. Clinically, the possibility of developing a false lumen certainly exists when the lesion is occluded or pseudo-occluded and the true lumen is not obvious; or when the artery is so severely disrupted that it is impossible to determine whether the lumen is true or false. A previous case series of CAD also described difficulty securing the true lumen [14], and a case series involving aortic dissection reported failure in securing the true lumen in 5/23 (21.7%) cases [8].

Very few reported cases of CAS were performed in the false lumen; to our knowledge, only three reports of CAS were performed in the false lumen [3,5,11]. The cases in which CAS was performed in the false lumen, including the present case, are listed in Table 1.

**Table 1:** Reported cases of carotid artery stenting in the false lumen of acute cervical carotid artery dissection. M: male; F: female; AIS: acute ischemic stroke; N.A.: not assessed; mRS: modified Rankin scale

References	Year of publication	Age	Sex	Type	Lesion site	Reason for false lumen passage	Stent	Follow	Outcome
Befera et al. [3]	2019	48	F	AIS	Cervical	Total occlusion	Wallstent, Precise	3 months	mRS 1
Limbucci et al. [5]	2017	46	F	AIS	Tandem lesion	Total occlusion	Wallstent x2	3 months	mRS 1
Cohen et al. [11]	2011	35	M	iatrogenic	Cervical	True lumen collapse	Wingspan x2	N.A.	N.A.
Present case		49	F	AIS	Tandem lesion	Total occlusion	Wallstent x2	6 months	mRS 1

Of these four cases, the case by Cohen et al. [11] unavoidably performed CAS in the false lumen because the true lumen was delineated but could not be accessed even after multiple trials. In the other two cases [3,5], as well as our case, the true lumen was not delineated because of complete or pseudo-occlusion of the lesion, and the true lumen was inaccessible. No postoperative complications were reported in all four cases; the outcome was good in three cases, with an mRS score of 1 (the outcome was not assessed in one case [11]).

There are also few reports on false lumen stenting for acute arterial dissection in other organs. In the case of acute dissection of a coronary artery with complete occlusion, a stent was placed in the true-false-true lumen to achieve reopening, but one year later, the patient developed angina due to reopening of the true lumen and stent occlusion in the false lumen [15]. Another report mentioned a case of acute dissection of the main left coronary artery in which a stent was placed in the false lumen. The lumen failed to reopen, and the dissecting lumen extended to the distal left coronary artery, resulting in the patient’s death [16]. Moreover, in aortic dissection, a case of intestinal necrosis due to occlusion of a branch from the true lumen after stent misplacement in the false lumen, as well as occlusion of the stent two weeks after implantation, has been reported [8].

In cases where the true lumen cannot be secured due to chronic total occlusion (CTO) of a coronary artery or peripheral arterial lesion, reopening through the false lumen has been performed and established as a technique [17,18]. However, perforation (7%) and stent occlusion in the chronic phase (35%) have been reported as complications in coronary CTO [19]. In contrast, in peripheral arteries, puncture site hematoma, perforation, and distal embolization have been reported to occur in 8-17% of cases [19]. These reports state that stenting in the false lumen should be performed when it is difficult to secure the true lumen [17-19].

A few CAS procedures for CTO have also been reported in the carotid artery, and in some of these cases, the procedures may have been performed via a false lumen; however, no apparent complications have been reported [20].

Overall, the above-reported cases indicate that stent placement in the false lumen is associated with the possibility of stent occlusion after implantation, perforation, distal embolization, and distal progression of dissection. However, all these risks are based on the reports on other organs and chronic dissection. In all four acute CAD cases including the present case, no complications were observed [3,5,11]. Therefore, the above-assumed risks do not seem to apply directly to acute CAD because of the different nature of the lesion and the vascular characteristics.

No method of reliably securing the true lumen during revascularization procedures for CAD has been reported. In particular, when the lesion is completely occluded, it is impossible to recognize the true lumen; as in this case, the lumen that was thought to be the true lumen may turn out to be a false lumen. The most critical stage is at the beginning of the procedure because dilating the false lumen further narrows down the true lumen and makes it difficult to pass through.

Murata et al. described a method wherein a catheter was placed in the ipsilateral ECA for contrast agent injection. Subsequently, to depict the true lumen, a balloon-guiding catheter was placed to occlude the ICA and aspirate the contrast agent flowing into the distal ICA from the collateral vessels between the ECA and the ICAs in a retrograde fashion [21]. Several other methods have been reported to confirm the accessed lumen, such as the passage of a micro-guidewire through the lesion using three-dimensional digital subtraction angiography [12], IVUS [13] to determine the relationship between the wire and the lumen in CAD, and intravascular optical coherence tomography to confirm the lumen in coronary artery dissection [22].

Only Murata et al.’s method [21] can confirm the true lumen without accessing the lesion, and because both methods are relatively complex, they may not be suitable when reopening the intracranial artery, when the treatment should be applied as quickly as possible.

If a wire/catheter has entered the false lumen in a situation that requires rapid reopening, it is preferable to reestablish the true lumen. However, expanding a false lumen may be worth considering if it is difficult to secure the true lumen.

When stents are implanted by dilating the false lumen, issues may occur regarding (1) securing the distal true lumen, (2) balloon dilation, and (3) stent placement.

Regarding securing the distal true lumen, in the report of acute CAD, except for the case by Cohen et al. [11], in which another stent was placed distally before the procedure, reentry was achieved at the transition from the cranial entry point in all three cases. Limbucci et al. suggest that this is caused by the difference in fixation of the cervical and intracranial ICA [5]. For this anatomical reason, distal advancement of the guidewire may secure a true lumen, but if a true lumen cannot be secured beyond the petrous portion, the procedure should be terminated because it may progress to an intracranial artery dissection leading to a subarachnoid hemorrhage.

Balloon dilation is performed over the entire false lumen, including the entry and reentry point after creation of the reentry, if the entire lesion to be stented is not clearly delineated from the proximal true lumen to the distal true lumen. This technique is often performed in CTOs of coronary and peripheral arteries [17,18]. Cohen et al. performed a similar procedure with a 2-mm-diameter balloon [11]. We used a 3-mm-diameter balloon, with the intention of preventing rupture of the false lumen, which may be more fragile than usual, by using a balloon that is obviously smaller than the ordinary ICA. This time, we obtained a sufficient dilation to access the intracranial artery without obvious complications.

Stenting appears to be an essential procedure to avoid re-occlusion risk if the false lumen is dilated. The distal true lumen should be secured and deployed to cover the false lumen completely from the distal true lumen to the proximal true lumen. Furthermore, if multiple stents are used, they should be connected by a telescope [14].

However, this article has several limitations. CAS in the false lumen has few reported cases [3,5,11], and its efficacy and especially its long-term safety remain unclear. Several complications, such as the extension of the dissection lumen [16], vessel perforation, and stent reocclusion, have been reported when stenting in the false lumen is performed in other organs [18], and expansion of the false lumen is considered as an alternative technique when the true lumen cannot be secured in other organ diseases [17-19]. Therefore, CAS in a false lumen should be used for acute CAD only when the true lumen cannot be secured, and immediate revascularization is necessary. Further case studies are needed to clarify this hypothesis.

## Conclusions

This report describes a case of acute ischemic stroke caused by complete occlusion of the cervical ICA due to dissection and associated intracranial embolic occlusion, which was treated with false lumen stenting and thrombectomy. Although false lumen dilatation in CAD has traditionally been considered undesirable, the present findings and previous reports suggest that false lumen stenting for acute CAD may be a less dangerous procedure than previously thought. However, because of the paucity of reported cases and uncertainties regarding efficacy and safety, CAS in the false lumen should be indicated only when immediate recanalization is necessary and the true lumen cannot be secured.
